# *Mycobacterium bovis* Isolates with *M. tuberculosis* Specific Characteristics

**DOI:** 10.3201/eid1205.050200

**Published:** 2006-05

**Authors:** Tanja Kubica, Rimma Agzamova, Abigail Wright, Galimzhan Rakishev, Sabine Rüsch-Gerdes, Stefan Niemann

**Affiliations:** * National Reference Center for Mycobacteria, Borstel, Germany;; † National Center for Tuberculosis Problems, Almaty, Kazakhstan;; ‡ World Health Organization, Geneva, Switzerland

**Keywords:** tuberculosis, Mycobacterium bovis, Kazakhstan, resistance, dispatch

## Abstract

Our study is the first report of exceptional *Mycobacterium bovis* strains that have some characteristics of *M. tuberculosis*. The strains were isolated from 8 patients living in Kazakhstan. While molecular markers were typical for *M. bovis*, growth characteristics and biochemical test results were intermediate between *M. bovis* and *M. tuberculosis*.

*Mycobacterium bovis* causes tuberculosis (TB) mainly in cattle but has a broad host range and causes disease similar to that caused by *M. tuberculosis* in humans (*1*). It belongs to the *M. tuberculosis* complex (MTBC) that comprises the closely related human pathogens *M. tuberculosis* and *M. africanum* (*2*). Identification of *M. bovis* traditionally has been based on clear-cut differences in phenotypic characteristics and biochemical properties when compared to the other members of the MTBC (*1**,**2*). *M. bovis* shows a dysgonic colony shape on Lowenstein-Jensen medium, is negative for niacin accumulation and nitrate reduction, is susceptible to thiophene-2-carboxylic acid hydrazide (TCH), and shows microaerophilic growth on Lebek medium (*1**–**3*). A further criterion used for differentiation is the intrinsic resistance to pyrazinamide, which is found in most *M. bovis* isolates (*1**–**3*). In contrast, *M. tuberculosis* shows eugonic growth, is positive for niacin accumulation and nitrate reduction, is resistant to TCH, shows aerophilic growth on Lebek medium, and is usually not monoresistant to pyrazinamide (*2**,**3*).

More recently, several molecular methods have been developed that provide clear criteria for the identification of *M. bovis*. These comprise a variety of polymerase chain reaction (PCR) methods, e.g., based on DNA sequence variations in the direct repeat region of MTBC complex strains (spoligotyping [4]) or on single nucleotide polymorphisms (SNPs) in either the *oxyR* gene (*5*) or the *gyrB* gene (*6*). Furthermore, MTBC isolates can be differentiated by large sequence polymorphisms or regions of difference (RD), and according to their distribution in the genome, a new phylogenetic scenario for the different species of the MTBC has been suggested (*7**–**9*). The presence or absence of particular deletions has been proposed as being discriminative, e.g., lack of TdB1 for *M. tuberculosis* or lack of RD12 for *M. bovis*.

In routine diagnostics, the combination of phenotypic characteristics and biochemical features is sufficient to differentiate clinical *M. bovis* isolates, and in general, the results obtained are unambiguous. However, here we describe the characteristics of 8 strains of the MTBC that showed an unusual combination of phenotypic and biochemical attributes of both *M. bovis* and *M. tuberculosis*. Molecular analyses confirmed the strains as *M. bovis*, which in part have phenotypic and biochemical properties of *M. tuberculosis*.

## The Study

During a previous investigation of 179 drug-resistant isolates from Kazakhstan (*10*), we determined the presence of 8 strains showing monoresistance to pyrazinamide. Kazakhstan is the largest of the central Asian republics and is divided regionally into 14 oblasts. The investigation was performed as part of a nationwide drug resistance survey conducted by the national TB program of Kazakhstan with assistance from the World Health Organization/International Union against Tuberculosis and Lung Disease Global Project in 2001. The subset of this survey investigated here (n = 158) represents 100% of strains resistant to isoniazid, rifampin, ethambutol, or streptomycin isolated in 9 of the 14 Kazakhstan oblasts during the study period; 21 samples had fungal contamination or showed no growth (*10*). All strains were isolated from sputum samples.

To further clarify if these strains were monoresistant *M. tuberculosis* or *M. bovis* isolates intrinsically resistant to pyrazinamide, we performed several routine diagnostic tests traditionally used for species differentiation (*6*). All strains showed eugonic growth characteristics on Lowenstein-Jensen slants and on bromcresol purple medium (Figure), which in general is typical for *M. tuberculosis*. However, on bromcresol purple medium, classic *M. tuberculosis* isolates induce a pH-dependent change of color from blue to yellow, which was not observed in these cases (Figure). Furthermore, all 8 isolates were positive for niacin accumulation, negative for nitrate reduction, susceptible to TCH, and showed aerophilic growth on Lebek medium. Considering all results, the 8 strains showed a combination of test results that did not allow a clear differentiation as *M. bovis* or as *M. tuberculosis* (Table 1). Such a combination of test results would apply best to *M. africanum*, a species from which more variable test results have been reported (*3*). However, this species was probably was not isolated because *M. africanum* strains are usually not monoresistant to pyrazinamide (*3*).

**Figure:**
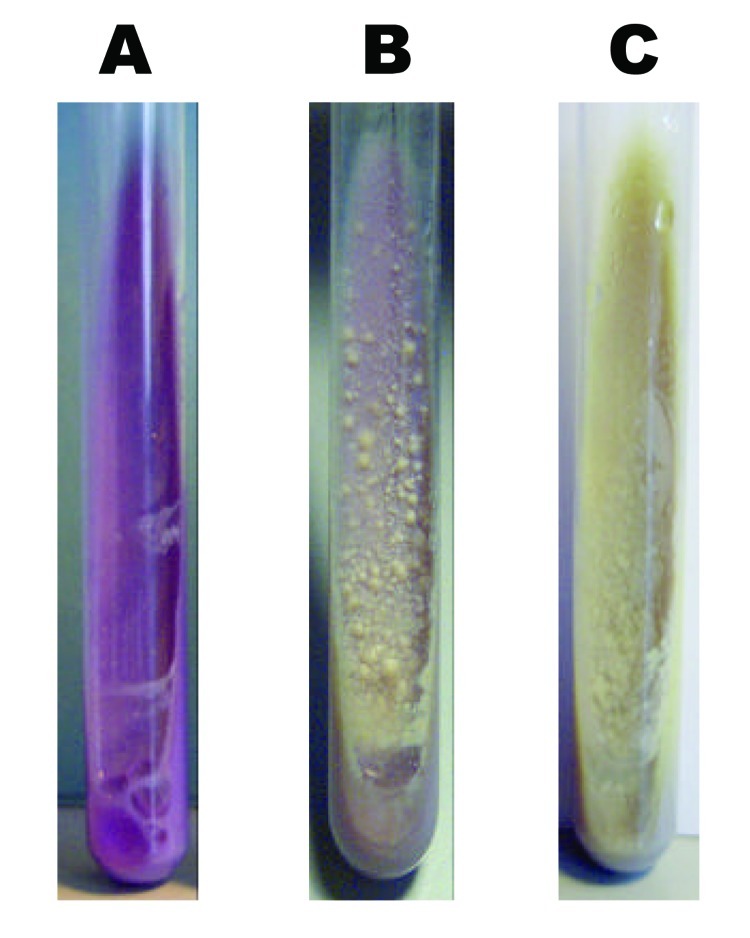
Growth morphology on bromcresol purple medium of *Mycobacterium bovis* (A), *M. tuberculosis* (C), and 1 of the strains analyzed (B).

**Table 1:** Phenotypic characteristics of type strains *Mycobacterium tuberculosis* H37 (ATCC 27294), *M. bovis* (ATCC19210), *M. africanum* (ATCC25420), and the strains analyzed*

Strains	Colony morphology†	Test result					
		TCH‡	PZA	Niacin accumulation	Nitrate reduction	Change of color of bromcresol medium	Growth on Lebek medium
Kazakhstan (n = 8)§	Eugonic	S	R	+	–	–	Aerophilic
ATCC *M. bovis*	Dysgonic	S	R	–	–	–	Microaerophilic
ATCC H37	Eugonic	R	S	+	+	+	Aerophilic
ATCC *M. africanum*	Dysgonic	S	S	+	+	–	Microaerophilic

*TCH, thiophene-2-carboxylic acid hydrazide; PZA, pyrazinamide; +, positive test result; –, negative test result; S, susceptible; R, resistant. †Growth characteristics on Lowenstein-Jensen and bromcresol purple slants. ‡Growth in presence of TCH. §All 8 strains showed identical test results.

Therefore, we investigated all strains with several molecular techniques previously used for differentiation within the MTBC (Table 2). They all had identical spoligotype patterns (hexcode 6B-57-5F-7F-FF-60, performed according to the methods of Kamerbeek et al. [4]), that lacked spacers 39–43 and identical IS*6110* DNA fingerprint patterns with 2 IS*6110* copies (data not shown, performed according to the methods of van Embden et al. [11]). All isolates carried the *M. bovis*–specific polymorphism in the *oxyR* gene (*5*), and none of them had the *M. bovis* BCG– specific deletion in the RD1 region (*12*). PCR analysis of other RDs (RD3, RD4, RD5, RD9, RD10, RD12, TbD1, and IS*1541*) showed results typical for *M. bovis* when compared with the RD signatures of the American Type Culture Collection strains of *M. tuberculosis*, *M. africanum*, and *M. bovis* (Table 2) and with previously published data (*7**,**8*). The intrinsic resistance to pyrazinamide was confirmed by DNA sequence analysis as all strains carried the *M. bovis*–specific single point mutation at nucleotide position 169 of the *pncA* gene.

**Table 2:** Genetic characteristics of type strains *Mycobacterium tuberculosis* H37 (ATCC 27294), *M. bovis* (ATCC19210), *M. africanum* (ATCC25420), and the strains analyzed*

Strains	Test result										
	TbD1	RD1	RD3	RD4	RD5	RD9	RD10	RD12	*IS1561*	*oxyR†*	*gyrB‡*
Kazakhstan (n = 8)§	1	1	1	0	0	0	0	0	1	1	*M. bovis*
*M. bovis* ATCC	1	1	1	0	0	0	0	0	1	1	*M. bovis*
*M. tuberculosis* H37	0	1	1	1	1	1	1	1	1	0	*M. tuberculosis*
*M. africanum* ATCC	1	1	0	1	1	0	0	1	1	0	*M. africanum*

*RD, region of difference; 0, region deleted; 1, region present. †Presence of *oxyR* mutation G to A at position 285; 1, polymorphism present; 0, polymorphism not present. ‡Classification according to *gyrb*-polymerase chain reaction restriction fragment length polymorphism analysis (Niemann et al. [3]). §All 8 strains showed identical test results.

Seven of the 8 strains were isolated from 30- to 55-year-old men, and 1 strain was from a 72-year-old woman. All but 1 patient had a history of previous antituberculosis treatment, but none of the strains showed any further resistance (data not shown). The patients originated from the oblast of Kostanajskaya in north Kazakhstan. Among all patients, no direct epidemiologic links could be established. However, 3 of the patients lived in the city of Kostanaj, while 5 came from rural areas. Before 1950, the Kazakh Steppe was a broad, continuous belt of grassland that stretched from the Ural River to the Altai foothills, covering large parts of Kostanajskaya; after the 1950s, the region was used extensively for agriculture. Information on contact with animals is not available, since cattle herds are only kept privately.

## Conclusions

We describe 8 strains of *M. bovis* with exceptional phenotypic characteristics that are intermediate between *M. tuberculosis* and *M. bovis*. This fact initially complicated a clear species differentiation; however, the battery of molecular tests performed clearly confirmed all strains as *M. bovis*. These tests included the presence of characteristic single nucleotide polymorphisms as well as an RD profile that is typical for the *M. bovis* lineage of the MTBC (*6**,**7*). To our knowledge, this is the first report describing *M. bovis* isolates with phenotypic characteristics and biochemical properties of *M. tuberculosis*. In our previous investigation of 176 *M. bovis* strains from Germany, all strains had phenotypic characteristics typical of *M. bovis*, and no strains similar to the isolates from Kazakhstan could be identified (*13*). The same result applies for the spoligotype patterns, as none of the strains in our database had an identical spoligotype pattern (data not shown). A further comparison with the international *M. bovis* spoligotype database (available from http://www.mbovis.org/spoligodatabase) identified 1 strain isolated in Argentina with an identical spoligotype pattern; however, no further information about his strain is available.

Whether the 8 strains analyzed represent strains of an ancestral phylogenetic lineage of *M. bovis* that might have been conserved because of the geographic isolation of that region of Kazakhstan or whether they gained their special characteristics by new mutations is a question that cannot be answered by the data obtained in this study. All strains have been isolated from humans. We cannot say if we have found an exceptional outbreak of a particular *M. bovis* strain or if the patients were infected directly by wildlife, livestock, or food, and the disease developed by chance during the study. However, an overall percentage of ≈5% of all resistant strains investigated in this study indicates that these isolates may be important in Kazakhstan. This also poses the question of whether these strains might become more virulent in humans if they acquired phenotypic/biochemical characteristics usually observed exclusively in *M. tuberculosis*. However, to address this question more precisely, longitudinal studies on the population structure of MTBC isolates in Kazakhstan obtained from humans and animals, in combination with experiments in virulence model systems, will be necessary. In any case, these strains represent ideal model organisms for analyzing the nature of the biologic differences observed between *M. bovis* and *M. tuberculosis*. To ensure a clear differentiation from other *M. bovis* strains, we suggest the name *M. bovis* subtype "Almaty" for this genotype. Almaty is the former capital and largest city of Kazakhstan.
